# Predictors of chronic kidney disease in type 1 diabetes: a longitudinal study from the AMD Annals initiative

**DOI:** 10.1038/s41598-017-03551-w

**Published:** 2017-06-12

**Authors:** Pamela Piscitelli, Francesca Viazzi, Paola Fioretto, Carlo Giorda, Antonio Ceriello, Stefano Genovese, Giuseppina Russo, Pietro Guida, Roberto Pontremoli, Salvatore De Cosmo

**Affiliations:** 1 Department of Medical Sciences, Scientific Institute “Casa Sollievo della Sofferenza”, San Giovanni Rotondo (FG), Italy; 2 Università degli Studi and IRCCS Azienda Ospedaliera Universitaria San Martino-IST, Genova, Italy; 3 Department of Medicine, University of Padova, Padova, Italy; 4 Diabetes and Metabolism Unit ASL Turin 5, Chieri (TO), Italy; 5 Institut d’Investigacions Biomèdiques August Pii Sunyer (IDIBAPS) and Centro de Investigación Biomédicaen Red de Diabetes y Enfermedades Metabólicas Asociadas (CIBERDEM), Barcelona, Spain; 6 U.O. Diabetologia e Malattie Metaboliche, Multimedica IRCCS, Sesto San Giovanni, Milano, Italy; 7 Department of Clinical and Experimental Medicine, University of Messina, Messina, Italy; 8 Associazione Medici Diabetologi, Rome, Italy

## Abstract

We evaluated 2,656 patients with type 1 diabetes mellitus and preserved renal function from the database of the Italian Association of Clinical Diabetologists network to identify clinical predictors for the development of chronic kidney disease. We measured estimated glomerular filtration rate (eGFR), urinary albumin excretion, HbA1c, lipids, blood pressure. Over a 5-year period 4.3% (n = 115) developed reduced eGFR (<60 ml/min/1.73 m^2^), 18.0% (n = 477) albuminuria, and 21.0% (n = 559) either one of the renal endpoints (CKD). Odds ratios for eGFR below 90 mL/min/1.73 m2 (1.48, P < 0.001), HbA1c (1.13, P = 0.002), triglycerides (1.04, P = 0.021 by 20 mg/dL), low density lipoprotein cholesterol (LDL-c) (0.95, P = 0.002 by 10 mg/dL) were independently related to the onset of CKD. Known duration of diabetes (1.15, P = 0.014 by 10 years), HbA1c (1.16, P = 0.001), triglycerides (1.05, P = 0.005 by 20 mg/dL), LDL-c (0.95, P = 0.003 by 10 mg/dL), antihypertensive treatment (2.28, P = 0.018) were related to the onset of albuminuria while age and presence of baseline eGFR values between 90 and 60 mL/min/1.73 m2, independently affected the developing of reduced eGFR (OR 1.95, P < 0.001 by 10 years and 2.92, P < 0.001). Patients with type 1 diabetes mellitus and unfavorable CV risk profile are at high risk of developing CKD. The two main traits of CKD share several determinants, although with some specificities.

## Introduction

Diabetes is one of the largest health emergencies of the 21^st^ century. The International Diabetes Federation Diabetes Atlas estimated that in 2015, there were 415 million patients with diabetes worldwide and by 2040 this figure will rise to 642 million people. Type 1 diabetes is less common, accounting for 7–12% of the total cases and it is still increasing by approximately 3% each year globally, particularly among children^1^.

Long-term complications due to diabetes are a major cause of disability, reduced quality of life and premature death^2^. Approximately 5.0 million people aged between 20 and 79 years died from diabetes in 2015, which accounts for 14.5% of global all-cause mortality among people in this age group^1^. This risk excess seems to be essentially driven by kidney disease^3^.

Chronic kidney disease (CKD) is detected clinically by screening for persistent increased urine albumin excretion and for a decreased estimated glomerular filtration rate (eGFR)^4^. Diabetes CKD is the leading cause of end-stage renal disease in the Western world^5^ and it is associated with an increased risk for cardiovascular (CV) events^6^, which in turn remain the leading cause of death in patients with type 1 diabetes mellitus^3,7,8^.

In addition to genetic determinants^9^, hyperglycemia, dyslipidemia, increased blood pressure (BP) and smoking are known to be risk factors for the onset and progression of diabetic renal damage^10,11^.

The natural history of diabetic nephropathy in patients with type 1 diabetes mellitus has traditionally been associated with an increase in urinary albumin excretion rate (AER), which is the first sign of renal damage and may foster progression to macroalbuminuria and later on decrease in GFR^12^. More recently however, several studies have provided a more heterogeneous picture of renal phenotype in T1D patients with CKD, with a significant number of patients progressing towards ESRD in the absence of albuminuria. Whether renal lesions found in non albuminuric CKD are specifically due to diabetes or may in part recognize different pathogenetic mechanisms is sometimes difficult to ascertain in clinical practice as renal biopsies are not routinely performed in most of these patients.

We retrospectively analysed a large cohort of patients with type 1 diabetes mellitus and normal renal function at baseline, to evaluate predictors for the development and progression of depressed kidney function + /− and/or albuminuria or its single components, and their relationship with traditional risk factors.

## Results

### Clinical features of study population at baseline

The main clinical features of the study population (n = 2,656) at baseline, are summarized in Table 1. Overall, the mean age was 44 ± 14 years, 56% of patients were males and the mean duration of diabetes was 17 ± 12 years. The average BMI was 24.4 ± 3.4 Kg/m2, indicating that the majority of patients had normal body weight. The glycemic control was rather unfair, mean values of HbA1c being 7.7% (60.66 mmol/mol), with about 70% of patients showing HbA1c values above 7% (53.0 mmol/mol). On the contrary, lipids and BP control were on average fairly good, with mean values of low density lipoprotein cholesterol (LDL-c) and BP of 110 mg/dL and 125/76 mmHg, respectively (Table 1). Baseline eGFR was 90 ± 16 mL/min/1.73 m2. Twenty percent of patients were receiving antihypertensive treatment (with a mean of 1.6 drugs per patient), and 18.2% were taking an ACE-I or an ARB. Retinopathy, either background (BG) or proliferative (PR), was more frequent among patients with CKD and with each of its components, particularly among patients with albuminuria (Table 1).

**Table 1:** Baseline clinical characteristics of whole population and divided by the occurrence of 5-year renal outcome among 2,656 patients with type 1 diabetes mellitus.

	All	CKD		p	eGFR <60 mL/min/1.73 m^2^		p	Albuminuria		p
	Patients	No	Yes		No	Yes		No	Yes	
	n = 2656	n = 2097	n = 559		n = 2541	n = 115		n = 2179	n = 477	
Male sex	1487 (56.0%)	1178 (56.2%)	309 (55.3%)	0.220	1437 (56.6%)	50 (43.5%)	0.979	1213 (55.7%)	274 (57.4%)	0.158
Age (years)	44 ± 14	43 ± 13	48 ± 15	<0.001	43 ± 13	60 ± 13	<0.001	43 ± 13	46 ± 15	0.024
Known duration of diabetes (years)	17 ± 12	16 ± 11	19 ± 12	<0.001	16 ± 11	23 ± 13	0.013	16 ± 11	19 ± 12	<0.001
BMI (Kg/m^2^)	24.4 ± 3.4	24.3 ± 3.3	24.6 ± 3.7	0.295	24.3 ± 3.3	25.3 ± 3.9	0.071	24.3 ± 3.3	24.5 ± 3.8	0.376
Serum creatinine (mg/dL)	0.84 ± 0.16	0.84 ± 0.16	0.85 ± 0.17	0.379	0.84 ± 0.16	0.92 ± 0.17	<0.001	0.84 ± 0.16	0.84 ± 0.17	0.351
eGFR (mL/min/1.73 m2)	99 ± 16	100 ± 16	95 ± 18	<0.001	100 ± 16	78 ± 14	<0.001	99 ± 16	98 ± 17	0.611
Serum uric acid (mg/dL)	3.8 ± 2.2	3.8 ± 2.4	3.9 ± 1.2	0.890	3.8 ± 2.2	4.0 ± 1.4	0.955	3.8 ± 2.3	3.9 ± 1.2	0.691
SUA in the top gender-specific quintile	275 (18.8%)	204 (17.7%)	71 (22.8%)	0.521	253 (18.1%)	22 (32.4%)	0.494	216 (18.0%)	59 (22.5%)	0.302
HbA1c (%) (mmol/mol)	7.7 ± 1.3 (60 ± 9)	7.6 ± 1.3 (59 ± 9)	7.9 ± 1.4 (63 ± 8)	<0.001	7.6 ± 1.3 (59 ± 9)	8.1 ± 1.5 (65 ± 7)	0.023	7.6 ± 1.3 (59 ± 9)	7.9 ± 1.5 (63 ± 7)	<0.001
HbA1c ≥7% (≥53 mmol/mol)	1843 (69.4%)	1433 (68.3%)	410 (73.3%)	0.187	1754 (69.0%)	89 (77.4%)	0.550	1495 (68.6%)	348 (73.0%)	0.189
Total cholesterol (mg/dL)	189 ± 34	189 ± 34	189 ± 35	0.248	189 ± 34	194 ± 35	0.591	189 ± 34	188 ± 35	0.309
Triglycerides (mg/dL)	84 ± 56	82 ± 54	93 ± 64	0.001	84 ± 57	91 ± 52	0.702	82 ± 54	94 ± 66	<0.001
Triglycerides ≥150 mg/dl	191 (7.2%)	137 (6.5%)	54 (9.7%)	0.015	181 (7.1%)	10 (8.7%)	0.867	141 (6.5%)	50 (10.5%)	0.001
HDL (mg/dL)	62 ± 19	62 ± 19	62 ± 18	0.101	62 ± 19	65 ± 18	0.431	63 ± 19	61 ± 18	0.059
HDL <40 M <50 F mg/dL	294 (11.1%)	227 (10.8%)	67 (12.0%)	0.229	282 (11.1%)	12 (10.4%)	0.896	235 (10.8%)	59 (12.4%)	0.205
LDL (mg/dL)	110 ± 31	111 ± 31	108 ± 31	0.064	110 ± 31	111 ± 31	0.949	111 ± 31	108 ± 31	0.058
LDL ≥100 mg/dL	1650 (62.1%)	1320 (62.9%)	330 (59.0%)	0.064	1583 (62.3%)	67 (58.3%)	0.437	1367 (62.7%)	283 (59.3%)	0.099
Systolic BP (mmHg)	125 ± 17	124 ± 16	129 ± 19	<0.001	125 ± 17	137 ± 18	<0.001	125 ± 17	128 ± 18	0.001
Diastolic BP (mmHg)	76 ± 9	75 ± 9	76 ± 9	0.024	76 ± 9	78 ± 9	0.035	76 ± 9	76 ± 9	0.071
Blood Pressure ≥140/85 mmHg	779 (29.3%)	569 (27.1%)	210 (37.6%)	<0.001	716 (28.2%)	63 (54.8%)	<0.001	610 (28.0%)	169 (35.4%)	0.002
*Retinopathy*										
Non-proliferative	495 (18.6%)	363 (17.3%)	132 (23.6%)	0.008	468 (18.4%)	27 (23.5%)	0.347	384 (17.6%)	111 (23.3%)	0.024
Proliferative	152 (5.7%)	102 (4.9%)	50 (8.9%)	<0.001	138 (5.4%)	14 (12.2%)	0.062	110 (5.0%)	42 (8.8%)	<0.001
Smokers	357 (26.2%)	271 (25.0%)	86 (31.0%)	0.004	349 (26.8%)	8 (13.6%)	0.207	278 (24.6%)	79 (34.2%)	0.001
Lipid-lowering treatment	416 (15.7%)	295 (14.1%)	121 (21.6%)	0.001	385 (15.2%)	31 (27.0%)	0.168	314 (14.4%)	102 (21.4%)	0.001
Treatment with statins	400 (15.1%)	281 (13.4%)	119 (21.3%)	<0.001	369 (14.5%)	31 (27.0%)	0.073	300 (13.8%)	100 (21.0%)	<0.001
Treatment with fibrates	9 (0.3%)	8 (0.4%)	1 (0.2%)	0.366	9 (0.4%)	0 (0.0%)	1.000	8 (0.4%)	1 (0.2%)	0.614
Antihypertensive treatment	534 (20.1%)	357 (17.0%)	177 (31.7%)	<0.001	480 (18.9%)	54 (47.0%)	<0.001	390 (17.9%)	144 (30.2%)	<0.001
Treatment with ACE-Is/ARBs	484 (18.2%)	324 (15.5%)	160 (28.6%)	<0.001	434 (17.1%)	50 (43.5%)	<0.001	355 (16.3%)	129 (27.0%)	<0.001
Aspirin	184 (6.9%)	128 (6.1%)	56 (10%)	0.024	165 (6.5%)	19 (16.5%)	0.141	140 (6.4%)	44 (9.2%)	0.026
Insulin pump	162 (6.1%)	132 (6.3%)	30 (5.4%)	0.564	155 (6.1%)	7 (6.1%)	0.420	139 (6.4%)	23 (4.8%)	0.238

Mean ± SD or absolute frequency (percentage). Serum uric acid in the top gender-specific quintile: >3.8 mg/dL in females and >5 mg/dL in males. CKD, chronic kidney disease; eGFR, estimated glomerular filtration rate; BMI, body mass index; SUA, serum uric acid; HbA1c, glycated haemoglobin; HDL, high-density lipoprotein cholesterol; LDL, low-density lipoprotein cholesterol; ACE-Is, angiotensin converting enzyme-inhibitors; ARBs, angiotensin II receptor antagonists. The p value refers to the association between patients’ characteristics and outcome at mixed logistic regression model adjusting for baseline eGFR. Patients’ baseline missing data: BMI in 131 (4.9%), serum uric acid in 1192 (44.9%), total cholesterol in 21 (0.8%), and smoking status in 1294 (48.7%).

By study design, all patients had normal urine albumin excretion and eGFR ≥60 mL/min/1.73 m2.

### Five-year follow-up outcomes

Over a 5-year follow-up period, a total of 21.0% of patients (n = 559) developed CKD, 4.3% (n = 115) developed reduced eGFR (i.e. eGFR <60 mL/min/1.73 m2) and 18.0% (n = 477) albuminuria (444 = microalbuminuria and 33 = macroalbuminuria). Only a minority of patients (n = 33, 1.2%) developed both features of chronic kidney disease (CKD). Baseline clinical features of patients grouped based on achieved renal outcome within the study period are also reported in Table 1. On average, patients who went on to develop CKD or any one of its components (i.e. eGFR <60 ml/min/1.73 m2 or albuminuria) showed a worse clinical and metabolic profile. They were older, with a longer history of diabetes and poorer glycemic control. They also showed higher systolic blood pressure (SBP) mean values and higher proportion of BP values above 140/85 mmHg, despite a greater prevalence of antihypertensive treatment, especially with an ACE-I or an ARB. Higher levels of triglycerides were associated with CKD and, particularly, with the development of albuminuria (Table 1). As expected, patients who went on to develop reduced eGFR also had lower eGFR at baseline (78 vs. 100 mL/min/1.73 m2, patients who developed reduced eGFR vs. patients who remained with stable eGFR, respectively) (Table 1).

In Table 2 we report risk estimation and the proportion of patients developing depressed eGFR and/or albuminuria stratified by age and duration of diabetes.

**Table 2:** 5-year incidence of CKD, low eGFR and albuminuria by age and duration of diabetes.

	N	Age	Duration	CKD		eGFR <60 mL/min/1.73 m^2^		Albuminuria	
		(years)	(years)	Incidence	Odds Ratio (95% CI)	Incidence	Odds Ratio (95% CI)	Incidence	Odds Ratio (95% CI)
*Age*									
18–30 years	440	25 ± 3	10 ± 7	80 (18.2%)	—	1 (0.2%)	—	80 (18.2%)	—
31–40 years	711	35 ± 3	14 ± 9	109 (15.3%)	0.71 (0.51–0.99) **p = 0.047**	8 (1.1%)	4.64 (0.58–37.40) p = 0.149	102 (14.3%)	0.65 (0.46–0.92) **p = 0.014**
41–50 years	672	45 ± 3	17 ± 11	129 (19.2%)	1.18 (0.88–1.59) p = 0.265	20 (3.0%)	2.53 (1.10–5.82) **p = 0.029**	114 (17.0%)	1.08 (0.79–1.47) p = 0.629
>50 years	833	60 ± 7	23 ± 13	241 (28.9%)	1.68 (1.29–2.20) **p < 0.001**	86 (10.3%)	3.59 (2.14–6.01) **p < 0.001**	181 (21.7%)	1.34 (1.00–1.78) **p = 0.047**
*Duration of diabetes*									
≤10 years	939	39 ± 13	5 ± 3	158 (16.8%)	—	26 (2.8%)	—	139 (14.8%)	—
11–20 years	841	42 ± 13	15 ± 3	171 (20.3%)	1.21 (0.94–1.57) p = 0.147	31 (3.7%)	1.10 (0.62–1.97) p = 0.738	150 (17.8%)	1.25 (0.95–1.64) p = 0.107
21–30 years	515	46 ± 12	25 ± 3	125 (24.3%)	1.20 (0.91–1.59) p = 0.204	29 (5.6%)	1.18 (0.67–2.09) p = 0.566	103 (20.0%)	1.19 (0.88–1.60) p = 0.250
>30 years	361	56 ± 11	38 ± 6	105 (29.1%)	1.06 (0.76–1.47) p = 0.744	29 (8.0%)	0.71 (0.40–1.28) p = 0.258	85 (23.5%)	1.19 (0.83–1.69) p = 0.345

Mean ± SD and absolute frequency (percentage). CI = Confidence Interval. Odds Ratio with 95% confidence interval of each category in comparison to the previous one. Odds Ratios for age were adjusted for duration of diabetes and vice versa. The bold value refers to statistical significance (p < 0.05).

The relationship between the onset of depressed kidney function + /− albuminuria and its traits and traditional cardiovascular risk factors was further investigated by multivariate logistic analysis (Table 3). Age independently affected the development of reduced eGFR, with a 10-year risk increase of 95%, (i.e. roughly a double of the risk), while duration of disease independently predicted albuminuria onset with a 10-year risk increase of 1.5%. In our setting gender did not arise as an independent risk factor for CKD or its traits.

**Table 3:** Multivariate analysis for the occurrence of 5-year renal outcome.

	CKD	p	eGFR<60 mL/min/1.73 m2	p	Albuminuria	p
	Odds Ratio (95% CI)		Odds Ratio (95% CI)		Odds Ratio (95% CI)	
Male sex	1.01 (0.81–1.27)	0.913	0.96 (0.59–1.56)	0.873	1.04 (0.82–1.32)	0.760
Age (by 10 years)	1.07 (0.96–1.18)	0.203	**1.95 (1.57–2.43)**	**<0.001**	0.93 (0.84–1.04)	0.227
Known duration of diabetes (by 10 years)	1.10 (0.99–1.21)	0.081	0.98 (0.81–1.18)	0.807	**1.15 (1.03–1.28)**	**0.014**
BMI (Kg/m^2^)						
27–29.9	0.87 (0.63–1.19)	0.369	0.85 (0.45–1.62)	0.623	0.92 (0.66–1.28)	0.628
≥30	1.02 (0.65–1.59)	0.941	1.57 (0.72–3.42)	0.252	1.04 (0.65–1.66)	0.862
eGFR below 90 by 10 mL/min/1.73 m^2^	**1.48 (1.26–1.73)**	**<0.001**	**2.92 (2.28–3.74)**	**<0.001**	1.08 (0.91–1.29)	0.389
HbA1c (%)	**1.13 (1.05–1.23)**	**0.002**	1.13 (0.94–1.36)	0.194	**1.16 (1.06–1.26)**	**0.001**
Triglycerides (by 20 mg/dL)	**1.04 (1.01–1.08)**	**0.021**	1.00 (0.92–1.09)	0.955	**1.05 (1.02–1.09)**	**0.005**
HDL (by 10 mg/dL)	0.95 (0.89–1.02)	0.160	1.00 (0.88–1.14)	0.994	0.97 (0.90–1.04)	0.321
LDL (by 10 mg/dL)	**0.95 (0.91–0.98)**	**0.002**	0.98 (0.91–1.05)	0.528	**0.95 (0.91–0.98)**	**0.003**
Systolic BP (by 10 mmHg)	1.06 (0.97–1.15)	0.176	1.07 (0.91–1.25)	0.430	1.05 (0.96–1.14)	0.305
Diastolic BP (by 10 mmHg)	1.00 (0.87–1.16)	0.963	1.06 (0.79–1.43)	0.692	1.01 (0.87–1.18)	0.900
*Retinopathy*						
Non-proliferative	1.12 (0.85–1.46)	0.426	0.95 (0.55–1.65)	0.862	1.09 (0.82–1.46)	0.548
Proliferative	**1.57 (1.03–2.38)**	**0.036**	1.31 (0.62–2.76)	0.475	**1.57 (1.01–2.44)**	**0.045**
Lipid-lowering treatment	1.09 (0.81–1.46)	0.567	0.81 (0.47–1.40)	0.455	1.22 (0.90–1.66)	0.208
Antihypertensive treatment	1.87 (0.97–3.61)	0.061	1.21 (0.37–4.00)	0.753	**2.28 (1.15–4.51)**	**0.018**
Treatment with ACE-Is/ARBs	0.93 (0.48–1.80)	0.822	1.20 (0.36–4.01)	0.763	0.77 (0.38–1.53)	0.455
Aspirin	0.84 (0.56–1.27)	0.416	0.75 (0.38–1.47)	0.397	0.93 (0.60–1.44)	0.741
Insulin pump	0.96 (0.61–1.51)	0.846	2.45 (0.99–6.02)	0.052	0.75 (0.46–1.25)	0.275
*Complete case analysis*						
Serum uric acid (mg/dL)	0.96 (0.87–1.06)	0.417	0.98 (0.85–1.14)	0.810	0.97 (0.87–1.07)	0.515
Smokers	**1.69 (1.21–2.36)**	**0.002**	0.88 (0.37–2.11)	0.777	**1.84 (1.29–2.62)**	**0.001**

See Table 1 legend for abbreviations.

### Effects of clinical characteristics

Worse glycemic control affected the onset of albuminuria, with an increased risk of 16% for every 1% increase in HbA1c, but it does not appear to bear clinical relevance with regards to the risk of developing low eGFR. The presence of antihypertensive treatment was strongly and directly related to the onset of albuminuria but not eGFR reduction.

Among patients with baseline eGFR values between 90 and 60 mL/min/1.73 m2, the risk of developing CKD or reduced eGFR was increased by a measure of 48 to 192%, respectively each for 10 ml/min/1.73 m2 eGFR reduction.

CKD and albuminuria, but not reduced eGFR, onset were also independently predicted by higher levels of triglycerides with 4% and 5% increases risk for CKD and albuminuria respectively, each 20 mg/dl of serum triglycerides (Table 3).

**Figure 1:**
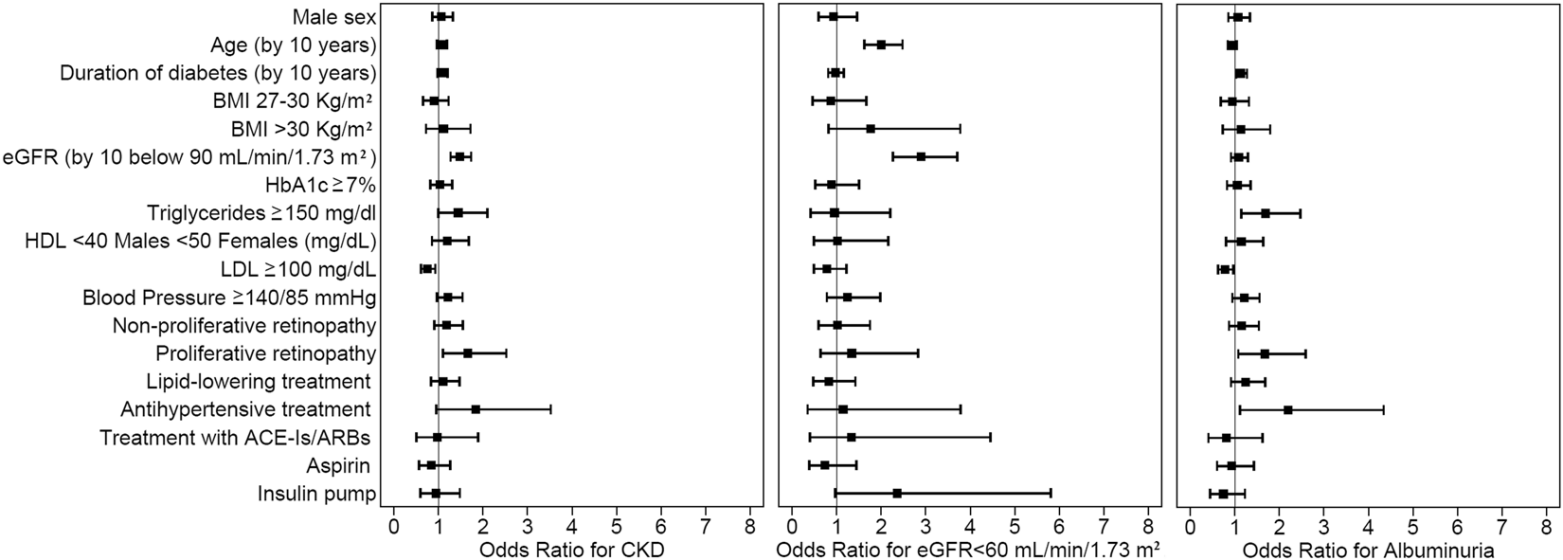
Association of baseline characteristics (categorical traits) with the risk of having CKD, eGFR <60 mL/min/1.73 m2 or albuminuria. Data are expressed as odds ratios (ORs) and 95% confidence intervals (95% CIs).

Figure 1 shows the association of study variables (categorical traits) with the risk of having CKD, low eGFR or albuminuria. In particular, when we considered HbA1c value as a categorical variable (i.e. above or below 7%), the above mentioned relationship with CKD and its components was no longer statistically significant.

**Figure 2:**
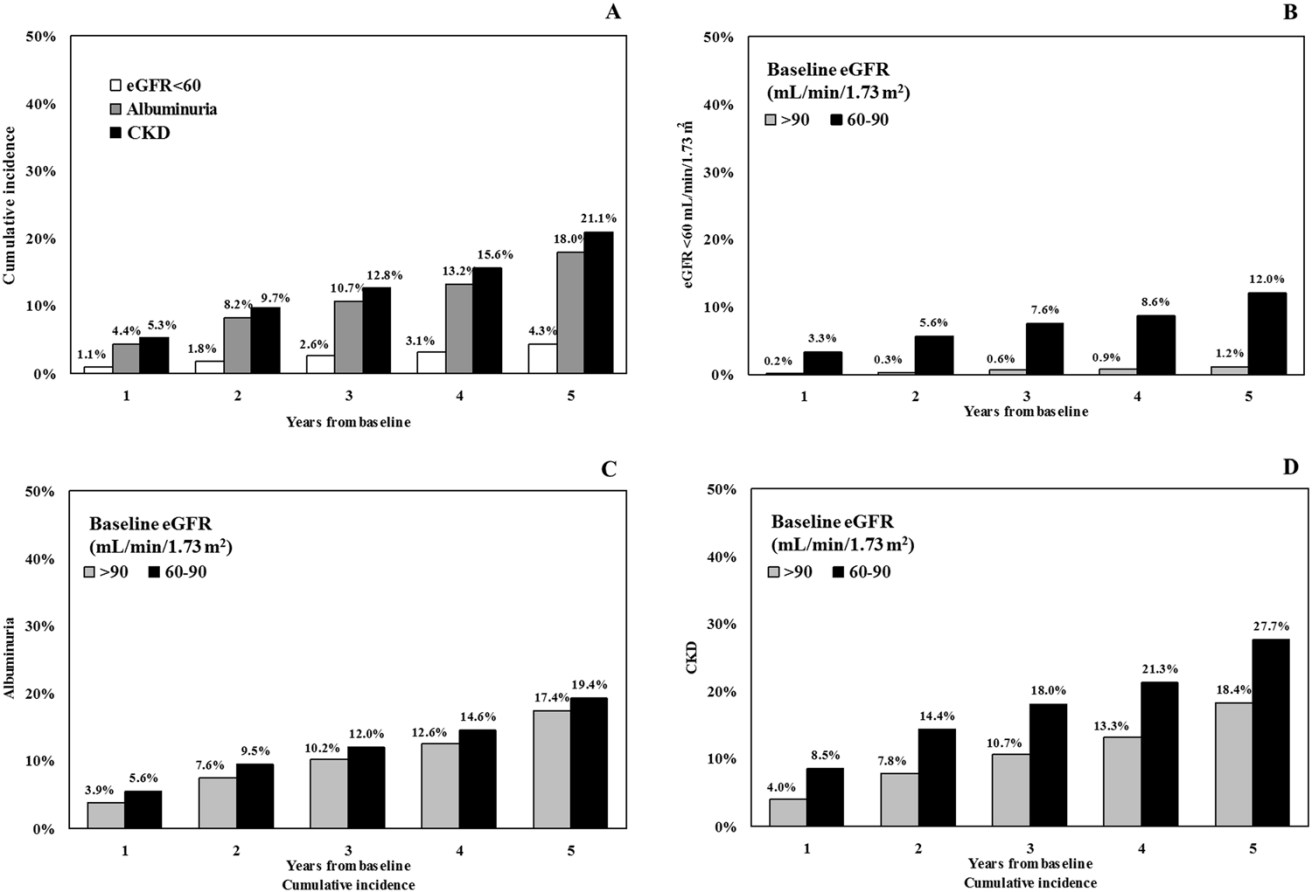
Proportion by year of patients with estimated glomerular filtration rate (eGFR) <60 mL/min/1.73 m2, albuminuria and CKD at the 5-year post baseline assessments (Panel A). Proportion by year of patients with estimated glomerular filtration rate (eGFR) <60 mL/min/1.73 m2, at the 5-year post baseline assessments according to baseline eGFR < or >90 mL/min/1.73 m2 (Panel B). Proportion by year of patients with albuminuria, at the 5-year post baseline assessments according to baseline eGFR < or >90 mL/min/1.73 m2 (Panel C). Proportion by year of patients with CKD at the 5-year post baseline assessments according to baseline eGFR < or >90 mL/min/1.73 m2 (Panel D).

The cumulative incidence of CKD, reduced eGFR (i.e., eGFR <60 mL/min/1.73 m2) and albuminuria in the whole population or divided according to eGFR values >90 or <90 and >60 mL/min/1.73 m2 is reported in Fig. 2, panels A, B, C, D. As expected, the incidence of renal outcomes increased progressively over the 5-year follow-up period and was higher among patients with a lower eGFR at baseline (i.e., 60–90 mL/min/1.73 m2).

### Tree analysis models

Finally, by applying a tree analysis model we identified 5 patient subgroups at different risk for developing eGFR <60 mL/min/1.73 m2 with the strongest variable in differentiating the risk being baseline eGFR value. In fact, patients with baseline eGFR ≥75 mL/min/1.73 m2, younger age (i.e. <65 years) and shorter duration of diabetes (i.e. <28 years) showed the lowest incidence of eGFR <60 mL/min/1.73 m2. When this subgroup of patients is considered as the reference class (class 5), the incidence of low eGFR progressively increases moving from class 5 to class 1, whose patients have a lower eGFR and age >65years (Supplemental Fig. 2 and Supplemental Table 3).

As for albuminuria, 5 different subgroups were identified by tree analysis model. The strongest variable in differentiating the risk of developing albuminuria was the presence of antihypertensive treatment. In detail, patients without antihypertensive treatment with triglycerides ≤85/dl and HbA1c <8.7% (71.59 mmol/mol) had the lowest incident rate of albuminuria (12.7%). When this subgroup of patients is considered as the reference class, the incidence of albuminuria progressively increases from class 5 to class 1 (i.e. rate = 44.1%), whose patients were on antihypertensive treatment and had eGFR <80 mL/min/1.73 m2. (Supplemental Table 3).

## Discussion

This is the first survey from Italy investigating the natural history of kidney dysfunction in a large sample of adult patients with type 1 diabetes mellitus, providing important hints regarding clinical risk factors which predict CKD and its traits, in a real life setting.

Our data show that in a type 1 diabetes mellitus population with a mean age of 44 years and duration of diabetes of 17 years, up to 21.0% of patients (n = 559) developed CKD (i.e. eGFR <60 mL/min/1.73 m2 or albuminuria), 4.3% (n = 115) reduced eGFR (i.e. eGFR <60 mL/min/1.73 m2) and 18.0% (n = 477) albuminuria (i.e. 444 microalbuminuria and 33 macroalbuminuria), over a 5-year follow-up period. We can estimate that, in Italy, in 5 year follow-up at least about one out of five type 1 diabetes mellitus patients attending diabetes centers will develop CKD.

There was a constant and progressively greater incidence of renal events during the study period with an average yearly rate of reduced eGFR or albuminuria onset between 0.5 and 1.2% and between 2.5 and 4.8%, respectively. It is worth nothing that lower baseline eGFR values significantly affected the rate of renal impairment more than albuminuria onset.

As for the effect of age and duration of diabetes on CKD development, it appears that while the prevalence of CKD steadily increases along with age and duration of disease, the risk of developing CKD is relatively unaffected by duration of diabetes. The relative constant incidence of CKD with increasing disease duration could be due to a number of confounding factors, such as the influence of genetic predisposition to develop renal complication as well as a selection bias due to premature death in those prone to develop renal disease.

Overall, in our setting, more patients developed albuminuria than reduced eGFR, a finding in line with previously published longitudinal^13^ and cross-sectional studies^14^.

The incidence of CKD we report here is in accord with the trend in prevalence recently reported by Murphy *et al*. in the NHANES survey and significantly higher than what is observed in the general population making diabetes mellitus, both type 1 and type 2, in its own right a remarkable risk factor for CKD^15^.

The cumulative incidence of CKD traits we describe here, namely reduced eGFR and albuminuria, is similar to that reported by DCCT/EDIC study. In the DCCT/EDIC study 6% of patients developed low eGFR (i.e <60 mL/min/1,73 m2), 16% microalbuminuria, 9% macroalbuminuria. Although our study had a relatively short follow-up, the mean age of our study population and the long duration of disease at baseline of patients we recruited make our results comparable to those from DCCCT/EDIC^13^.

The cumulative incidence of CKD in patients with type 1 diabetes mellitus appears to be decreasing over time. The figure we describe herein is substantially lower when compared to data from earlier epidemiological studies carried out in the 80 s which suggested that up to 45% of type 1 diabetes mellitus patients developed macroalbuminuria^16^. The significant improvement in the management of traditional risk factors, namely glycaemia, BP and cholesterol, as well as the reduction in the rate of cigarette smoking and increased use of renin-angiotensin system inhibitors recorded over the years, may account for these differences.

Whereas renal impairment and albuminuria in patients with type 1 diabetes mellitus shared a number of risk factors, there also was a distinct set of variables, which predicted one but not the other. These results support the concept that albuminuria and renal impairment may not necessarily reflect the same underlying pathophysiological mechanism in type 1 diabetes mellitus. Notwithstanding, some peculiarities need to be mentioned. First of all, in our setting, worse glycemic control appears to play a significant role in increasing the risk of developing CKD, with some specificity. In fact, while in the univariate model increased HbA1c associated with CKD risk and each of its components, in the multivariate model we found a 13% greater risk for CKD and a 16% greater risk for albuminuria onset for each 1% increase in HbA1c. We could not detect any significant association between glycemic control and reduced eGFR. This finding clearly supports the strong role of glycemic control as a risk factor for albuminuria in type 1 diabetes mellitus patients with a minor role for renal impairment. A weak relationship between glucose control and GFR decline has also been reported by other observational and intervention studies such as the DCCT study^17^. On the other hand, the role of hyperglycemia as an established risk factor for albuminuria onset results also from several intervention studies^18^, including DCCT^19^, which unquestionably demonstrate that intensive glucose control results in a clinically important and durable reduction in the risk of albuminuria onset the role of glycemic control on the progression of kidney damage is not so clear.

Triglycerides also are strongly related to albuminuria onset but appear to have no significant effect on reduced eGFR. This finding has also been reported by Hadjadi *et al*. who described serum triglycerides as a predictive factor for the development and the progression of renal and retinal complications in patients with type 1 diabetes mellitus^20^. Moreover, low levels of triglycerides were associated with regression of microalbuminuria^21^.

Patients who went on to develop CKD and its components had a significantly higher baseline systolic BP as compared to those who remained with stable kidney function. However, in our study, the deleterious effect of blood pressure disappeared in the multivariate model. This somewhat unexpected finding could be explained, at least in part, by the optimal BP control (i.e. mean BP = 125 ± 17/76 ± 9 mmHg) recorded in our study patients. Nonetheless, the presence (and degree) of antihypertensive treatment entails a more than two-fold risk of albuminuria onset. Antihypertensive treatment could therefore be considered as a proxy of arterial hypertension, which can be the real driver of kidney damage onset.

The presence of retinopathy, either BR or PR, was more common among patients who developed CKD.

After fully adjusted analysis, PR remained strongly associated to albuminuria, which developed fifty-seven percent more often in patients with baseline PR as compared to those without retinopathy. This association suggests common pathogenetic mechanisms for PR and albuminuria.

In contrast with results of previous studies^22^, we found no association between serum uric acid (SUA) levels and renal outcomes. This discrepancy may mainly be due to differences in the characteristics of the population studied, such as age and duration of disease, duration of follow up or difference in the definition of outcomes.

As expected, smoking was associated with albuminuria alone while we found no association between reduced eGFR and smoking. These findings is in line with the hypothesis proposed by Chuahirun *et al*., who found that the effect of smoking was mediated by albuminuria in patients with type 2 diabetes^23^.

Finally, the tree analysis allowed us to investigate also the interaction between the many clinical variables we considered and their hierarchical impact on the incidence of reduced eGFR or albuminuria. Results of these analyses may provide useful information for real life clinical practice. In fact, we found that among patients with type 1 diabetes mellitus and preserved renal function, those with ongoing antihypertensive treatment and eGFR <80 mL/min/1.73 m2 are at highest risk for albuminuria onset while those with baseline eGFR <75 mL/min/1.73 m2 and age >60 years are at highest risk for renal impairment.

Our study has some limitations as well as several strengths that should be mentioned. Among the first ones, we must acknowledge that the duration of follow-up is relatively short, although the mean age of patients (i.e. 44 years) and long duration of disease (i.e. 17 years) allowed us to record a significant number of events. Second, laboratory parameters, including serum creatinine were not measured in a single, centralized laboratory which may have led to some variability, especially in GFR estimation. In addition, information on albuminuria has been gathered only as a categorical trait and this may also have contributed to variability in the outcome measure. Moreover, data regarding the entire 5-year follow-up period were available for most but not all patients and therefore caution should be taken not to generalize our findings, as mortality from competitive risk was not positively collected in the missing subgroup. Baseline clinical features of the subgroup with missing values, however, were similar to that of the entire cohort. Finally, we did not screen our population for non-diabetic diseases predisposing to CKD development or for the use of nephrotoxic agents. However, considering the large number of patients recruited, their relative young age, the periodic visits they have had at diabetes centers and the extremely low incidence of non-diabetic kidney disease in this setting, we do not believe that non-diabetic diseases or nephrotoxic agents could have significantly affected our results.

On the other hand, we should mention the large size and homogeneous clinical characteristics of study sample as well as the representative geographical distribution of the recruiting centers, which certainly contribute to a good representation of real life clinical conditions.

In conclusion, our 5-year retrospective analysis of a large, real life, cohort of patients with type 1 diabetes mellitus and preserved kidney function at baseline showed a 21.0% cumulative incidence of CKD, with nearly 4% of patients developing renal impairment and 18% albuminuria. Our results also indicate that i. patients with type 1 diabetes mellitus and unfavorable CV risk profile are at high risk of developing CKD, ii. the two main traits of kidney dysfunction (i.e. reduced eGFR and albuminuria) in patients with type 1 diabetes mellitus share several determinants, although with some specificity that needs to be taken into account when describing the risk profile of our patients.

## Methods

### Study participants and design

In Italy diabetes care is mainly provided by a public network of about 700 diabetes clinics where a team of specialists provide diagnostic confirmation, prevention and treatment for diabetes and its complications through close patients follow-up and regular check-ups^24,25^.

In the present report we analyzed a large sample of patients with type 1 diabetes, (according to American Diabetes Association 2003 criteria) followed-up at 137 centers among those participating in the Italian Association of Clinical Diabetologists (Associazione Medici Diabetologi, AMD) initiative. The analysis was performed using the data set of electronic medical records collected between 1st January, 2004 and 30th June, 2011. For the purpose of the analysis, we considered only patients who were ≥18 years old and with a follow-up evaluation within 60 ± 6 months complete for data about estimated GFR (eGFR) and albuminuria. Of 5,486 patients identified, we excluded those with albuminuria, eGFR <60 mL/min/1.73 m2 or a previous eGFR value discordant (i.e. <60 mL min/1.73 m2) or those with missing data of antidiabetic treatment. Two-thousand-six-hundred- fifty-six patients from 118 clinics constitute the study population (Supplemental Fig. 1). The centers involved in the study are homogeneously distributed throughout the country.

### Data collection

As already reported, the analysis of the database is an attempt by the Italian Association of AMD initiative to identify a set of indicators that can be used in the context of continuous quality improvement. Participating centres adopted the same software systems for everyday management of outpatients, while a specially developed software package allowed us to extract the information we intended to analyze from all the clinical databases (AMD Data File). Moreover, data from all participating centers were collected and centrally analyzed anonymously^24,25^.

### Laboratory methods

This initiative includes measuring and monitoring HbA1c, blood pressure (BP), low-density lipoprotein (LDL-c), total and high density lipoprotein cholesterol (HDL-c) and triglycerides. The use of specific classes of drugs (insulin, statins and two or more anti-hypertensive agents) was also evaluated. Since normal ranges for HbA1c varied among centers, the percentage change with respect to the upper normal value (measured value⁄upper normal limit) was estimated and multiplied by 6.0 in order to allow comparisons among the centers. Kidney function was assessed by serum creatinine and urinary albumin excretion measurements. GFR was estimated for each patient using a standardized serum creatinine assay and the Chronic Kidney Disease Epidemiology Collaboration formula^26^.

Increased UAE was diagnosed as: i. microalbuminuria if urinary albumin concentration was >30 and ≤300 mg/l, or if UAE rate was >20 and ≤200 μg/min, or if urinary albumin-to-creatinine ratio (ACR) was >2.5 mg/mmol in men and >3.5 mg/mmol in women and ≤30 mg/mmol in both gender; ii. macroalbuminuria if urinary albumin concentration was >300 mg/l, or if UAE rate was >200 μg/min, or if ACR was >30 mg/mmol in both gender. Albuminuria indicates patients with either micro or macroalbuminuria.

### Measurements

At each participating centre all patients underwent physical examination and BP measurements according a standardized protocol. BP was measured with the patient in the sitting position after a 5-minute rest, with a mercury sphygmomanometer. Systolic BP and diastolic BP were read to the nearest 2 mmHg. Disappearance of Korotkoff sounds (phase V) was the criterion for diastolic BP. Three measurements were taken at 2-minute intervals and the average value was used to define clinical systolic BP and diastolic BP. CKD was defined as diabetes with albuminuria or low eGFR (i.e. <60 mL/min/1.73 m2) or both.

Information on the presence of diabetic retinopathy, BR or PR, was also available.

The outcomes were i. CKD (i.e. eGFR less than 60 mL/min/1.73 m2 and/or albuminuria), ii. eGFR less than 60 mL/min/1.73 m2 and iii. Albuminuria. Occurrence of pre specified endpoints was evaluated on a yearly basis over the 5-year study period. Patients were considered to have reached the study endpoints if, at any time during the study period, they met the above indicated criteria.

### Approval

In the AMD database, data can be linked together by a unique anonymous identifier that is encrypted to protect patients’ privacy. Because this automated system precludes identification of individual patients, according to the Italian law ethical committee approval and informed consent were not required. The results were internally approved by the AMD ANNALS Scientific Committee.

### Statistical analysis

Data are given as mean values ± standard deviation (SD); categorical variables are described as frequencies and percentages. The analysis was aimed to evaluate the associations between baseline characteristics with renal outcomes during the study period. A mixed logistic regression model with diabetes clinics fitted as random effect was used for each renal outcome to estimate odds ratios (ORs) with their 95% confidence interval (95% CI). Multivariate models over the entire population were fitted including a missing indicator variable for patients without BMI value. Multivariate models including serum uric acid and smoking status were performed separately as complete-case analysis for patients with all data available (1464 and 1362 patients, respectively). To derive a hierarchical tree of event risk, a logistic model for renal outcome (separately for eGFR <60 mL/min/1.73 m2 and albuminuria) was used to split the data recursively into subgroups selecting the variable with the minimum p-value (if significant <0.05). Continuous variables were analyzed for all values from the 5^th^ to 95^th^ percentile selecting the cut-point with the lowest p-value. The tree-building process was stopped after 3 iterations to obtain no more than 8 nodes. The analyses were made using STATA software, Version 14 (StataCorp, College Station, Texas). P values of <0.05 were considered statistically significant.

## Electronic supplementary material

Supplementary info

## References

[1] IDF Diabetes Atlas 7^th^ Edition International Diabetes Federation. http://www.idf.org/idf-diabetes-atlas-seventh-edition Visit November 5, 2016 (2015).

[2] Nathan DM (2013). DCCT/EDIC Research Group. Diabetes control and complications trial/epidemiology of diabetes interventions and complications study at 30 years: advances and contributions. Diabetes.

[3] Groop PH (2009). FinnDiane Study Group. The presence and severity of chronic kidney disease predict all-cause mortality in type 1 diabetes. Diabetes.

[4] National Kidney Foundation (2007). KDOQI clinical practice guidelines and clinical practice recommendations for diabetes and chronic kidney disease. Am J Kidney Dis.

[5] United States renal Data System USRDS annual data report 2015. National Institute of Diabetes and Digestive and Kidney Diseases, Bethesda, MD Chapter 1: Incidence, Prevalence, Patient Characteristics, and Treatment Modalities https://www.usrds.org/2016/view/v2_01.aspx Visit March 7, 2017 (2015).

[6] Valmadrid CT (2000). The risk of cardiovascular disease mortality associated with microalbuminuria and gross proteinuria in persons with older-onset diabetes mellitus. Arch Intern Med.

[7] Krolewsky AS (2015). Progressive Renal Decline: The New Paradigm of Diabetic Nephropathy in Type 1 Diabetes. Diabetes Care.

[8] Rossing P (2005). The changing epidemiology of diabetic microangiopathy in type 1 diabetes. Diabetologia.

[9] Pezzolesi MG (2009). DCCT/EDIC Research Group, Doria A, Rogus JJ, Rich SS, Warram JH, Krolewski AS. Genome-wide association scan for diabetic nephropathy susceptibility genes in type 1 diabetes. Diabetes.

[10] Hovind, P. *et al*. Predictors for the development of microalbuminuria and macroalbuminuria in patients with type 1 diabetes: inception cohort study. *BMJ* 328–1105 (2004). 10.1136/bmj.38070.450891.FE PMC406322 15096438

[11] Feodoroff, M. *et al*. Smoking and progression of diabetic nephropathy in patients with type 1 diabetes. *Acta Diabetol Dec* 14 (2015). 10.1007/s00592-015-0822-0 26668013

[12] Viberti GC (1982). Microalbuminuria as a predictor of clinical nephropathy in insulin-dependent diabetes mellitus. Lancet.

[13] DCCT/EDIC research group. (2014). Effect of intensive diabetes treatment on albuminuria in type 1 diabetes: long-term follow-up of the Diabetes Control and Complications Trial and Epidemiology of Diabetes Interventions and Complications study. Lancet Diabetes Endocrinol..

[14] de Boer IH (2011). MD Temporal Trends in the Prevalence of Diabetic Kidney Disease in the United States. JAMA..

[15] Murphy D (2016). Trends in Prevalence of Chronic Kidney Disease in the United States. Ann Intern Med..

[16] Hovind P (2004). Predictors for the development of microalbuminuria and macroalbuminuria in patients with type 1 diabetes: inception cohort study. BMJ..

[17] de Boer IH (2011). DCCT/EDIC Research Group. Intensive Diabetes Therapy and Glomerular Filtration Rate in Type 1 Diabetes. N Engl J Med..

[18] Feldt-Rasmussen B (1986). Effect of two years of strict metabolic control on progression of incipient nephropathy in insulin-dependent diabetes. Lancet.

[19] The Diabetes Control and Complications Trial Research Group The effect of intensive therapy of diabetes on the development and progression of long-term complications in insulin-dependent diabetes mellitus. *N Engl J Med*. **329**, 977–86 (1993). 10.1056/NEJM199309303291401 8366922

[20] Hadjadj S (2004). Serum triglycerides are a predictive factor for the development and the progression of renal and retinal complications in patients with type 1 diabetes. Diabetes Metab..

[21] Perkins BA (2003). Regression of microalbuminuria in type 1 diabetes. N Engl J Med. J.

[22] Hovind P (2009). Serum uric acid as a predictor for development of diabetic nephropathy in type 1 diabetes: an inception cohort study. Diabetes..

[23] Chuahirun T (2003). Cigarette smoking and increased urine albumin excretion are interrelated predictors of nephropathy progression in type 2 diabetes. Am J Kidney Dis.

[24] Nicolucci A (2010). Four-year impact of a continuous quality improvement effort implemented by a network of diabetes outpatient clinics: the AMD-Annals initiative. Diabet Med..

[25] De Cosmo S (2015). Serum Uric Acid and Risk of CKD in Type 2 Diabetes. Clin J Am Soc Nephrol..

[26] Levey AS (2009). A new equation to estimate glomerular filtration rate. Ann Intern Med..

