# Publisher Correction: Predictors of chronic kidney disease in type 1 diabetes: a longitudinal study from the AMD Annals initiative

**DOI:** 10.1038/s41598-018-23163-2

**Published:** 2018-04-12

**Authors:** Pamela Piscitelli, Francesca Viazzi, Paola Fioretto, Carlo Giorda, Antonio Ceriello, Stefano Genovese, Giuseppina Russo, Pietro Guida, Roberto Pontremoli, Salvatore De Cosmo

**Affiliations:** 1 Department of Medical Sciences, Scientific Institute “Casa Sollievo della Sofferenza”, San Giovanni Rotondo (FG), Italy; 2 Università degli Studi and IRCCS Azienda Ospedaliera Universitaria San Martino-IST, Genova, Italy; 3 Department of Medicine, University of Padova, Padova, Italy; 4 Diabetes and Metabolism Unit ASL Turin 5, Chieri (TO), Italy; 5 Institut d’Investigacions Biomèdiques August Pii Sunyer (IDIBAPS) and Centro de Investigación Biomédicaen Red de Diabetes y Enfermedades Metabólicas Asociadas (CIBERDEM), Barcelona, Spain; 6 U.O. Diabetologia e Malattie Metaboliche, Multimedica IRCCS, Sesto San Giovanni, Milano, Italy; 7 Department of Clinical and Experimental Medicine, University of Messina, Messina, Italy; 8 Associazione Medici Diabetologi, Rome, Italy

Correction to: *Scientific Reports* 10.1038/s41598-017-03551-w, published online 12 June 2017

The original version of this Article contained a typographical error in the spelling of the author Salvatore De Cosmo, which was incorrectly given as Salvatore De De Cosmo. This has now been corrected in the PDF and HTML versions of the Article.

