# Supplementary material for: Predictors of chronic kidney disease in type 1 diabetes: a longitudinal study from the AMD Annals initiative
Source: Sci Rep. 2017 Jun 12;7:3313. doi: 10.1038/s41598-017-03551-w (PMC5468280; doi:10.1038/s41598-017-03551-w)
Supplement: Supplementary file 1 — Supplementary info [file 41598_2017_3551_MOESM1_ESM.pdf]

# Supplementary Information for: Predictors of chronic kidney disease in type 1 diabetes: a longitudinal study from the AMD Annals initiative.

Authors: Pamela Piscitelli[1], Francesca Viazzi[2]; Paola Fioretto[3], Carlo Giorda[4]; Antonio Ceriello[5,6]; Stefano Genovese[6]; Giuseppina Russo[7]; Pietro Guida[8]; Roberto Pontremoli[2] and Salvatore De Cosmo[1].

[1]Department of Medical Sciences, Scientific Institute "Casa Sollievo della Sofferenza", San Giovanni Rotondo (FG), Italy; [2]Università degli Studi and IRCCS Azienda Ospedaliera Universitaria San Martino-IST, Genova, Italy; [3]Department of Medicine, University of Padova, Padova, Italy. [4]Diabetes and Metabolism Unit ASL Turin 5, Chieri (TO), Italy; [5]Institut d'Investigacions Biomèdiques August Pii Sunyer (IDIBAPS) and Centro de Investigación Biomédicaen Red de Diabetes y Enfermedades Metabólicas Asociadas (CIBERDEM), Barcelona, Spain; [6]U.O. Diabetologia e Malattie Metaboliche, Multimedica IRCCS, Sesto San Giovanni, Milano, Italy; [7]Department of Clinical and Experimental Medicine, University of Messina, Messina, Italy; [8]Associazione Medici Diabetologi, Rome, Italy.

**1. Fig. S1**: Flow diagram for selection of study patients.

**2. Fig. S2**: Figure that illustrates the hierarchical tree of glomerular filtration rate (eGFR) <60 mL/min/1.73m2 risk during follow-up.

**1-Supplemental Figure S1**

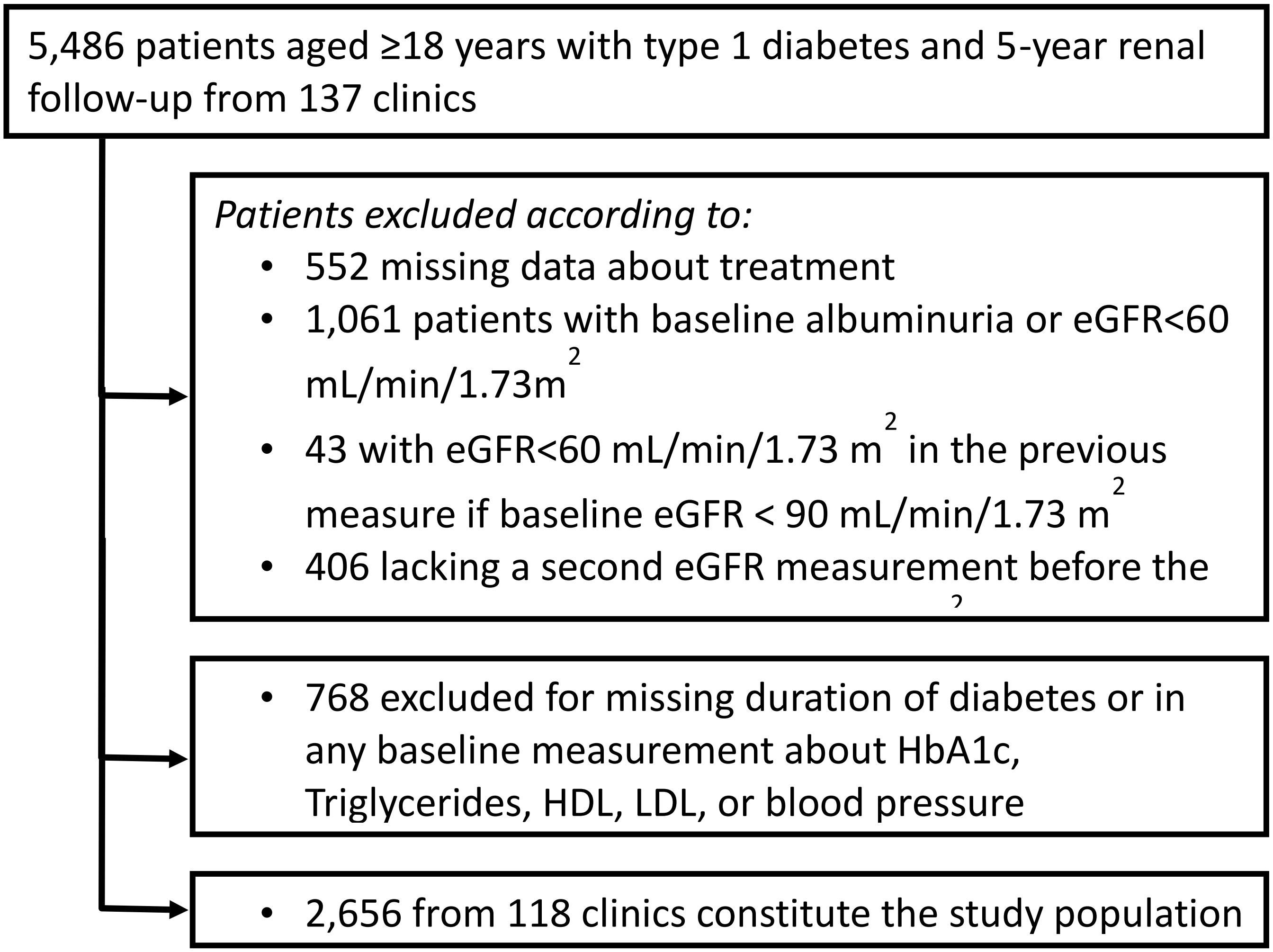

# 2-Supplemental Figure S2

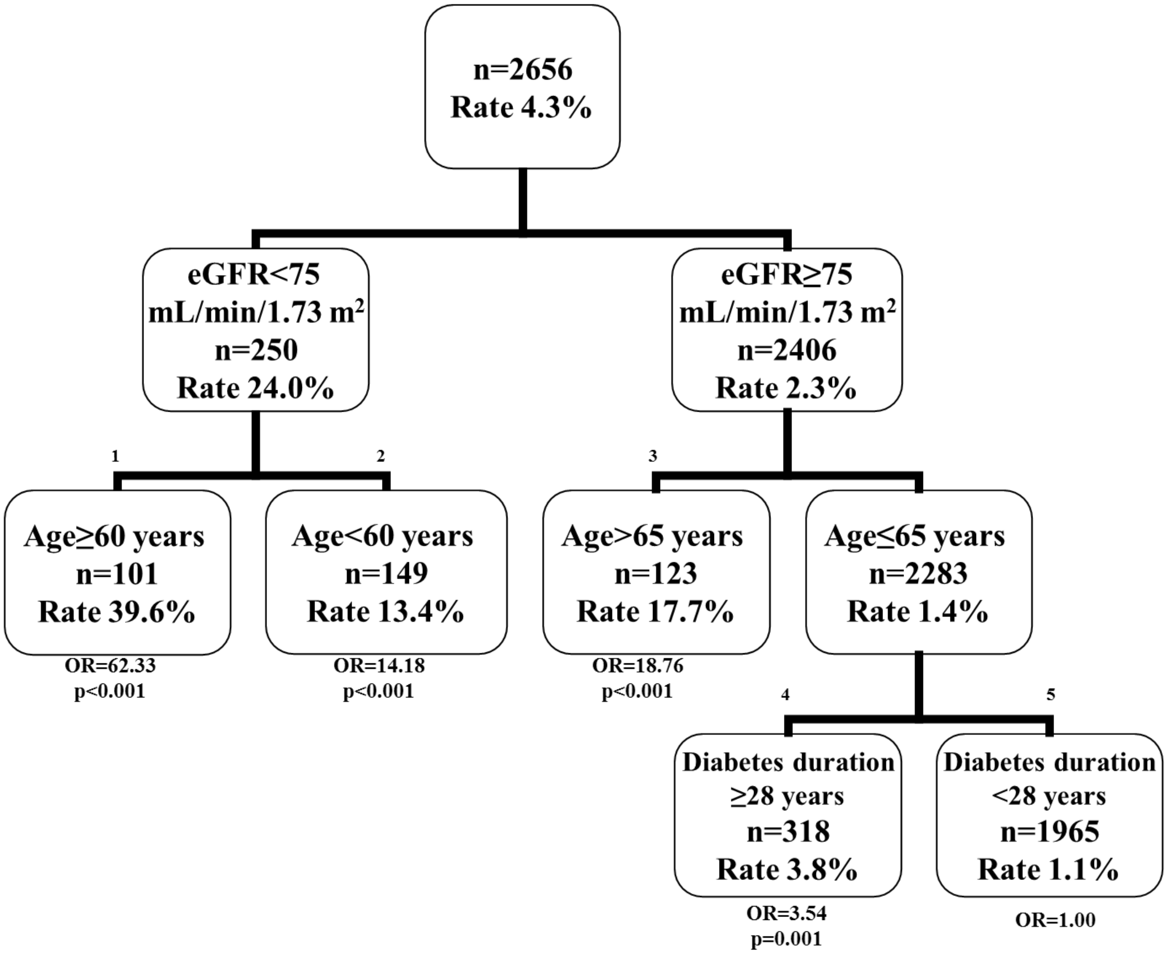

# Supplementary Tables

**Supplementary Table S1 Multivariate analysis for the occurrence of 5-year renal outcome.**

| | CKD | | eGFR <60 mL/min/1.73m2 | | Albuminuria | |
|---|---|---|---|---|---|---|
| | **Odds Ratio (95%CI)** | **p** | **Odds Ratio (95%CI)** | **p** | **Odds Ratio (95%CI)** | **p** |
| Male sex | 1.07 (0.86-1.32) | 0.560 | 0.93 (0.59-1.46) | 0.748 | 1.07 (0.85-1.34) | 0.560 |
| Age (by 10 years) | 1.07 (0.97-1.18) | 0.170 | **2.00 (1.62-2.48)** | **<0.001** | 0.93 (0.84-1.04) | 0.214 |
| Known duration of diabetes (by 10 years) | 1.09 (0.98-1.20) | 0.099 | 0.97 (0.81-1.16) | 0.725 | **1.14 (1.02-1.27)** | **0.018** |
| BMI (Kg/m$^2$) | | | | | | |
| 27-29.9 | 0.90 (0.66-1.22) | 0.491 | 0.87 (0.45-1.67) | 0.677 | 0.94 (0.68-1.31) | 0.737 |
| ≥30 | 1.11 (0.72-1.72) | 0.630 | 1.76 (0.82-3.78) | 0.148 | 1.14 (0.72-1.79) | 0.576 |
| eGFR below 90 by 10 mL/min/1.73 m2 | **1.48 (1.27-1.73)** | **<0.001** | **2.90 (2.26-3.71)** | **<0.001** | 1.09 (0.91-1.29) | 0.354 |
| HbA1c≥7% | 1.04 (0.82-1.31) | 0.765 | 0.88 (0.52-1.50) | 0.647 | 1.05 (0.82-1.35) | 0.674 |
| Triglycerides ≥150 mg/dl | 1.44 (0.99-2.10) | 0.056 | 0.95 (0.41-2.20) | 0.909 | **1.68 (1.15-2.47)** | **0.008** |
| HDL <40M <50F mg/dL | 1.20 (0.85-1.68) | 0.300 | 1.02 (0.48-2.15) | 0.959 | 1.15 (0.80-1.63) | 0.453 |
| LDL ≥100 mg/dL | **0.75 (0.60-0.93)** | **0.008** | 0.77 (0.49-1.22) | 0.269 | **0.77 (0.62-0.97)** | **0.026** |
| Blood Pressure ≥140/85 mmHg | 1.22 (0.96-1.54) | 0.101 | 1.24 (0.78-1.97) | 0.368 | 1.21 (0.94-1.55) | 0.133 |
| *Retinopathy* | | | | | | |
| Non-proliferative | 1.18 (0.90-1.54) | 0.228 | 1.01 (0.59-1.75) | 0.962 | 1.16 (0.87-1.54) | 0.316 |
| Proliferative | **1.66 (1.09-2.52)** | **0.017** | 1.34 (0.63-2.83) | 0.445 | **1.67 (1.08-2.59)** | **0.022** |
| Lipid-lowering treatment | 1.10 (0.83-1.47) | 0.503 | 0.82 (0.48-1.42) | 0.484 | 1.24 (0.91-1.68) | 0.172 |
| Antihypertensive treatment | 1.83 (0.95-3.52) | 0.070 | 1.15 (0.35-3.78) | 0.824 | **2.20 (1.11-4.34)** | **0.024** |
| Treatment with ACE-Is/ARBs | 0.97 (0.50-1.89) | 0.929 | 1.33 (0.40-4.45) | 0.641 | 0.81 (0.41-1.62) | 0.555 |
| Aspirin | 0.84 (0.56-1.27) | 0.409 | 0.74 (0.38-1.45) | 0.378 | 0.93 (0.60-1.43) | 0.728 |
| Insulin pump | 0.94 (0.60-1.48) | 0.792 | 2.36 (0.96-5.81) | 0.061 | 0.74 (0.45-1.23) | 0.244 |
| *Complete case analysis* | | | | | | |
| SUA in the top gender-specific quintile | 0.90 (0.62-1.30) | 0.563 | 1.00 (0.49-2.04) | 0.995 | 0.93 (0.63-1.38) | 0.728 |
| Smokers | **1.75 (1.25-2.44)** | **0.001** | 0.87 (0.36-2.09) | 0.757 | **1.89 (1.34-2.68)** | **<0.001** |

See Table 1 legend for abbreviations.

**Supplementary Table S2. Baseline clinical characteristics and missing information of type 1 diabetes mellitus patients included or excluded from the study.**

| | **Included** | **Excluded** | **Included** | **Excluded** |
|---|---|---|---|---|
| | **n=2656** | **n=768** | ***Missing, n (%)*** | ***Missing, n (%)*** |
| Male sex | 1487 (56.0%) | 447 (58.2%) | - | - |
| Age (years) | 44±14 | 42±13 | - | - |
| Known duration of diabetes (years) | 17±12 | 17±11 | - | 64 (8.3%) |
| BMI (Kg/m$^2$) | 24.4±3.4 | 24.7±3.5 | 131 (4.9%) | 398 (51.8%) |
| Serum creatinine (mg/dL) | 0.84±0.16 | 0.85±0.16 | - | - |
| eGFR (mL/min/1.73 m2) | 99±16 | 100±16 | - | - |
| Serum uric acid (mg/dL) | 3.8±2.2 | 3.7±1.2 | 1192 (44.9%) | 234 (30.5%) |
| Serum uric acid in the top gender-specific quintile | 275 (18.8%) | 99 (18.5%) | - | - |
| HbA1c (%) (mmol/mol) | 7.7±1.3(60±9) | 8.0±1.5(64±7) | - | 26 (3.4%) |
| HbA1c≥7% | 1843 (69.4%) | 566 (76.3%) | - | - |
| Total cholesterol (mg/dL) | 189±34 | 192±37 | 21 (0.8%) | 114 (14.8%) |
| Triglycerides (mg/dL) | 84±56 | 86±97 | - | 149 (19.4%) |
| Triglycerides ≥150 mg/dl | 191 (7.2%) | 39 (6.3%) | - | - |
| HDL (mg/dL) | 62±19 | 61±16 | - | 143 (18.6%) |
| HDL <40M <50F mg/dL | 294 (11.1%) | 70 (11.2%) | - | - |
| LDL (mg/dL) | 110±31 | 115±35 | - | 159 (20.7%) |
| LDL ≥100 mg/dL | 1650 (62.1%) | 425 (69.8%) | - | - |
| Systolic BP (mmHg) | 125±17 | 126±17 | - | 595 (77.5%) |
| Diastolic BP (mmHg) | 76±9 | 77±9 | - | 595 (77.5%) |
| Blood Pressure ≥140/85 mmHg | 779 (29.3%) | 52 (30.1%) | - | - |
| *Retinopathy* | | | | |
| Non-proliferative | 495 (18.6%) | 171 (22.3%) | - | - |
| Proliferative | 152 (5.7%) | 29 (3.8%) | - | - |
| Smokers | 357 (26.2%) | 52 (25.7%) | 1294 (48.7%) | 566 (73.7%) |
| Lipid-lowering treatment | 416 (15.7%) | 78 (10.2%) | - | - |
| Treatment with statins | 400 (15.1%) | 72 (9.4%) | - | - |
| Treatment with fibrates | 9 (0.3%) | 4 (0.5%) | - | - |
| Antihypertensive treatment | 534 (20.1%) | 60 (7.8%) | - | - |
| Treatment with ACE-Is/ARBs | 484 (18.2%) | 54 (7.0%) | - | - |
| Aspirin | 184 (6.9%) | 25 (3.3%) | - | - |
| Insulin pump | 162 (6.1%) | 49 (6.4%) | - | - |
| *5-year outcomes* | | | | |
| CKD | 559 (21.0%) | 120 (15.6%) | - | - |
| eGFR<60 mL/min/1.73 m2 | 115 (4.3%) | 34 (4.4%) | - | - |
| Albuminuria | 477 (18.0%) | 93 (12.1%) | - | - |

| | | | | |
|---|---|---|---|---|
| eGFR<60 and albuminuria | 33 (1.2%) | 7 (0.9%) | - | - |

See Table 1 legend for abbreviations.

**Supplementary Table S3. Baseline clinical characteristics of patients identified in nodes of hirarchical tree for glomerular filtration rate <60 mL/min/1.73 $m^2$ and for albuminuria.**

| | *Nodes of hirarchical tree for glomerular filtration rate <60 mL/min/1.73 $m^2$* | | | | | *Nodes of hierarchical tree for albuminuria* | | | | |
|---|---|---|---|---|---|---|---|---|---|---|
| | **1** | **2** | **3** | **4** | **5** | **1** | **2** | **3** | **4** | **5** |
| | **n=101** | **n=149** | **n=123** | **n=318** | **n=1965** | **n=137** | **n=397** | **n=669** | **n=216** | **n=1237** |
| Male sex | 46 (45.5%) | 47 (31.5%) | 68 (55.3%) | 164 (51.6%) | 1162 (59.1%) | 65 (47.4%) | 249 (62.7%) | 439 (65.6%) | 101 (46.8%) | 633 (51.2%) |
| Known duration of diabetes (years) | 26±15 | 20±12 | 26±14 | 35±6 | 12±8 | 27±14 | 21±12 | 15±10 | 15±10 | 15±11 |
| BMI (Kg/$m^2$) | 25.3±3.6 | 24.3±3.6 | 24.7±3.4 | 24.4±3.2 | 24.3±3.4 | 26.4±4.0 | 25.6±3.5 | 24.8±3.5 | 24.2±3.1 | 23.5±2.9 |
| Serum creatinine (mg/dL) | 0.99±0.13 | 1.05±0.12 | 0.76±0.13 | 0.81±0.15 | 0.83±0.16 | 0.99±0.14 | 0.80±0.15 | 0.86±0.16 | 0.81±0.17 | 0.83±0.16 |
| eGFR (mL/min/1.73m2) | 68±4 | 69±4 | 87±7 | 96±11 | 104±14 | 71±6 | 99±12 | 99±16 | 101±16 | 101±16 |
| Serum uric acid (mg/dL) | 4.3±1.5 | 3.9±1.2 | 3.6±1.0 | 4.0±3.3 | 3.8±2.1 | 4.4±1.3 | 4.1±2.7 | 4.0±2.7 | 3.4±0.9 | 3.7±1.8 |
| SUA in the top gender-specific quintile | 23 (42.6%) | 32 (30.5%) | 10 (14.7%) | 32 (20.4%) | 178 (16.5%) | 42 (48.8%) | 41 (19.7%) | 81 (21.1%) | 12 (9.8%) | 99 (14.9%) |
| HbA1c (%) | 8.1±1.1 | 7.7±1.2 | 7.9±1.1 | 7.7±1.3 | 7.6±1.4 | 8.1±1.2 | 7.7±1.2 | 7.8±1.4 | 9.7±1.0 | 7.1±0.9 |
| HbA1c (mmol/mol) | (65±11) | (60±10) | (86±11) | (60±9) | (59±8) | (65±10) | (60±10) | (61±8) | (82±12) | (54±13) |
| HbA1c≥7% | 87 (86.1%) | 107 (71.8%) | 101 (82.1%) | 234 (73.6%) | 1314 (66.9%) | 116 (84.7%) | 305 (76.8%) | 497 (74.3%) | 216 (100.0%) | 709 (57.3%) |
| Total cholesterol (mg/dL) | 194±37 | 196±35 | 192±31 | 195±30 | 187±35 | 192±34 | 191±34 | 200±38 | 187±31 | 183±31 |
| Triglycerides (mg/dL) | 96±42 | 86±46 | 79±29 | 78±37 | 85±61 | 92±42 | 91±68 | 133±73 | 61±13 | 59±14 |
| Triglycerides ≥150 mg/dl | 12 (11.9%) | 11 (7.4%) | 4 (3.3%) | 14 (4.4%) | 150 (7.6%) | 12 (8.8%) | 36 (9.1%) | 143 (21.4%) | 0 (0.0%) | 0 (0.0%) |
| HDL (mg/dL) | 65±21 | 64±17 | 67±21 | 66±19 | 61±18 | 63±19 | 60±20 | 57±19 | 67±18 | 65±18 |
| HDL <40M <50F mg/dL | 10 (9.9%) | 17 (11.4%) | 12 (9.8%) | 24 (7.5%) | 231 (11.8%) | 13 (9.5%) | 58 (14.6%) | 115 (17.2%) | 15 (6.9%) | 93 (7.5%) |
| LDL (mg/dL) | 111±36 | 114±31 | 108±28 | 114±28 | 109±31 | 111±30 | 113±31 | 117±34 | 108±29 | 106±28 |
| LDL ≥100 mg/dL | 58 (57.4%) | 104 (69.8%) | 75 (61.0%) | 223 (70.1%) | 1190 (60.6%) | 89 (65.0%) | 261 (65.7%) | 473 (70.7%) | 131 (60.6%) | 696 (56.3%) |
| Systolic BP (mmHg) | 141±17 | 128±16 | 140±17 | 130±18 | 123±16 | 138±16 | 134±17 | 125±16 | 124±16 | 122±16 |
| Diastolic BP (mmHg) | 77±9 | 76±9 | 77±10 | 75±9 | 76±9 | 77±9 | 79±10 | 76±9 | 76±9 | 74±8 |
| Blood Pressure ≥140/85 mmHg | 64 (63.4%) | 52 (34.9%) | 73 (59.3%) | 120 (37.7%) | 470 (23.9%) | 80 (58.4%) | 193 (48.6%) | 189 (28.3%) | 61 (28.2%) | 256 (20.7%) |
| Non-proliferative retinopathy | 22 (21.8%) | 32 (21.5%) | 36 (29.3%) | 100 (31.4%) | 305 (15.5%) | 45 (32.8%) | 101 (25.4%) | 112 (16.7%) | 44 (20.4%) | 193 (15.6%) |
| Proliferative retinopathy | 11 (10.9%) | 12 (8.1%) | 20 (16.3%) | 47 (14.8%) | 62 (3.2%) | 14 (10.2%) | 41 (10.3%) | 39 (5.8%) | 13 (6.0%) | 45 (3.6%) |
| Smokers | 5 (9.4%) | 11 (15.5%) | 10 (16.7%) | 38 (25.9%) | 293 (28.4%) | 7 (10.1%) | 52 (24.6%) | 127 (37.1%) | 23 (22.3%) | 148 (23.2%) |
| Lipid-lowering treatment | 31 (30.7%) | 29 (19.5%) | 46 (37.4%) | 70 (22.0%) | 240 (12.2%) | 60 (43.8%) | 147 (37.0%) | 92 (13.8%) | 14 (6.5%) | 103 (8.3%) |
| Treatment with statins | 29 (28.7%) | 29 (19.5%) | 45 (36.6%) | 66 (20.8%) | 231 (11.8%) | 57 (41.6%) | 142 (35.8%) | 85 (12.7%) | 14 (6.5%) | 102 (8.2%) |
| Treatment with fibrates | 0 (0.0%) | 0 (0.0%) | 0 (0.0%) | 2 (0.6%) | 7 (0.4%) | 2 (1.5%) | 0 (0.0%) | 6 (0.9%) | 0 (0.0%) | 1 (0.1%) |
| Antihypertensive treatment | 53 (52.5%) | 39 (26.2%) | 68 (55.3%) | 104 (32.7%) | 270 (13.7%) | 137 (100.0%) | 397 (100.0%) | 0 (0.0%) | 0 (0.0%) | 0 (0.0%) |
| Treatment with ACE-Is/ARBs | 50 (49.5%) | 35 (23.5%) | 60 (48.8%) | 94 (29.6%) | 245 (12.5%) | 124 (90.5%) | 360 (90.7%) | 0 (0.0%) | 0 (0.0%) | 0 (0.0%) |
| Aspirin | 24 (23.8%) | 12 (8.1%) | 32 (26.0%) | 43 (13.5%) | 73 (3.7%) | 44 (32.1%) | 79 (19.9%) | 21 (3.1%) | 3 (1.4%) | 37 (3.0%) |
| Insulin pump | 2 (2.0%) | 11 (7.4%) | 0 (0.0%) | 22 (6.9%) | 127 (6.5%) | 6 (4.4%) | 26 (6.5%) | 30 (4.5%) | 14 (6.5%) | 86 (7.0%) |

| | | | | | | | | | | |
|---|---|---|---|---|---|---|---|---|---|---|
| *5-year outcomes* | | | | | | | | | | |
| CKD | 58 (57.4%) | 45 (30.2%) | 46 (37.4%) | 76 (23.9%) | 334 (17.0%) | 70 (51.1%) | 107 (27.0%) | 155 (23.2%) | 43 (19.9%) | 184 (14.9%) |
| eGFR<60 mL/min/1.73 m2 | 40 (39.6%) | 20 (13.4%) | 21 (17.1%) | 12 (3.8%) | 22 (1.1%) | 38 (27.7%) | 16 (4.0%) | 26 (3.9%) | 4 (1.9%) | 31 (2.5%) |
| Albuminuria | 32 (31.7%) | 31 (20.8%) | 31 (25.2%) | 67 (21.1%) | 316 (16.1%) | 48 (35.0%) | 96 (24.2%) | 137 (20.5%) | 39 (18.1%) | 157 (12.7%) |
| eGFR<60 and albuminuria | 14 (13.9%) | 6 (4.0%) | 6 (4.9%) | 3 (0.9%) | 4 (0.2%) | 16 (11.7%) | 5 (1.3%) | 8 (1.2%) | 0 (0.0%) | 4 (0.3%) |

See Table 1 legend for abbreviations. Nodes are those identified in Supplemental Figure 2 and 3.
